# Characterization of prosthetic knees through a low-dimensional description of gait kinematics

**DOI:** 10.1186/s12984-023-01160-5

**Published:** 2023-04-13

**Authors:** Simone Ranaldi, Cristiano De Marchis, Mariano Serrao, Alberto Ranavolo, Francesco Draicchio, Francesco Lacquaniti, Silvia Conforto

**Affiliations:** 1 Department of Industrial Electronics and Mechanical Engineering, University Roma TRE, Rome, Italy; 2 Department of Engineering, University of Messina, Messina, Italy; 3 Department of Medico-Surgical Sciences and Biotechnologies, University of Rome Sapienza, Rome, Italy; 4 Rehabilitation Centre, Policlinico Italia, Rome, Italy; 5 Department of Occupational and Environmental Medicine, Epidemiology and Hygiene, INAIL, Rome, Italy; 6 Department of Systems Medicine and Centre of Space Biomedicine, University of Rome Tor Vergata, Rome, Italy; 7 Laboratory of Neuromotor Physiology, IRCCS Santa Lucia Foundation, Rome, Italy

## Abstract

The characterization of both limbs’ behaviour in prosthetic gait is of key importance for improving the prosthetic components and increasing the biomechanical capability of trans-femoral amputees. When characterizing human gait, modular motor control theories have been proven to be powerful in providing a compact description of the gait patterns. In this paper, the planar covariation law of lower limb elevation angles is proposed as a compact, modular description of prosthetic gait; this model is exploited for a comparison between trans-femoral amputees walking with different prosthetic knees and control subjects walking at different speeds. Results show how the planar covariation law is maintained in prostheses users, with a similar spatial organization and few temporal differences. Most of the differences among the different prosthetic knees are found in the kinematic coordination patterns of the sound side. Moreover, different geometrical parameters have been calculated over the common projected plane, and their correlation with classical gait spatiotemporal and stability parameters has been investigated. The results from this latter analysis have highlighted a correlation with several parameters of gait, suggesting that this compact description of kinematics unravels a significant biomechanical meaning. These results can be exploited to guide the control mechanisms of prosthetic devices based purely on the measurement of relevant kinematic quantities.

## Introduction

Walking with a unilateral lower limb prosthesis is a complex biomechanical task that requires a variety of different compensation mechanisms for the management of the asymmetries in the inertia and the capacity to generate torque on the two legs [1]. In general, human walking is the result of the interplay of different kinematic and kinetic patterns, generated by control mechanisms at the level of the neuromuscular system; therefore, a complete characterization of gait in pathological conditions typically requires the joint analysis of information of different nature [2–5]. Therefore, the introduction of compact indicators that are able to explain different aspects of human movement could be an important advancement in the field of human movement analysis. The current state-of-the-art for clinically relevant quantitative analysis of human gait relies on the evaluation of several different gait spatio-temporal parameters or stability indicators, such as the dynamic margin of stability [6]; despite the reliability and the simple mathematical definition of those parameters, their meaning is limited only to the characterization of the kinematics of movement, without any direct link with the control mechanisms that generate the patterns. The aforementioned parameters have been widely used for the description of prosthetic gait [2, 7, 8], showing the accuracy of a kinematic description in the characterization of specific differences between healthy subjects and prostheses users.

Aiming to build compact descriptions of the control of human movement, modularity concepts have been widely exploited in the literature, especially for describing muscular activity as the combination of the action of muscle synergies [9–11]. This approach has been used for the characterization of gait in trans-femoral amputees [12], showing how with a compact description of muscle coordination it is possible to extract meaningful information related to gait patterns abnormalities. A complementary way of analysing modular control strategies in gait is the investigation of the planar covariation law of elevation angles; this model has been proposed before as an evidence for the patterned modular control strategies of the movement kinematics in a variety of locomotion tasks in healthy subjects [13–16]. According to this theory, the elevation angles of the lower limb segments (i.e. the angle on the sagittal plane between the long axis of the segment and the vertical direction) are controlled in a modular fashion, resulting typically in a dimensionality reduction from the original 3-dimensional space (i.e. the space defined by the elevation angles of the thigh, shank and foot segments) to a 2-dimensional space typically referred to as the covariance plane [16]. While it can be argued that this dimensionality reduction is a direct result of biomechanical constraints [17], evidence has been given that this covariation law still applies when different tasks and different constraints are taken into account [13, 14]. Although this theory has been assessed before for investigating trans-femoral prosthetic gait [19], previous results focused on the analysis of the validity of the covariation law also for pathological subjects and on the properties of the covariance plane. Moreover, there is limited literature investigating the meaning of the projection of the elevation angles on the plane itself. Although a functional meaning has been ascribed to a particular rotation of the two projected components [19, 20], no previous studies have analysed the neuromechanical implications of these projections in characterizing pathological movement.

In this work, we investigated planar covariation law in prosthetic gait, with particular focus on the equivalence of the covariance plane for patients and healthy controls, under the hypothesis that a common covariation plane underlies the differences in the coordination of lower limb segments. We computed different parameters on the common projected plane, and investigated their biomechanical significance in terms of correlation with spatiotemporal gait parameters. The results presented in this work have been used to characterize different kind of prosthetic devices under this description, aiming to provide additional information about the kinematic features of motor control strategies that are not directly accessible by conventional analyses of the kinematic and kinetic curves, as well as to qualitatively investigate the capability of such a description in predicting relevant movement features. A preliminary test on a subset of these data have been in described [21], and it is here extended to include a thorough neuromechanical characterization of prosthetic gait.

## Methods

### Participants

For this study, 19 unilateral trans-femoral amputees were involved (age: 53 ± 13 years old, height: 175 ± 7, weight: 87 ± 14), as well as 12 control participants (age: 54 ± 9 years old, height: 175 ± 7 cm, weight: 76 ± 7 kg). Patients were subdivided into three main groups, depending on the kind of prostheses used: polycentric mechanical (FM, 5 subjects), electronic (Ottobock C-Leg, FE, 7 subjects) and bionic (Ottobock Genium, FB, 7 subjects) knees. All the patients suffered an amputation due to traumatic injury, and were able to move independently with their prosthetic device at the time of the gait analysis. All the patients were characterized by a K-Level of 4 and participated to the test with their usual prosthesis, that had been using for at least 1 year. The overall population under analysis is then composed of 12 controls walking at two speeds (control self-selected, CSS, and control slow, CSL, see below), 5 mechanical, 7 electronic and 7 bionic knee users. A detailed description of the patients population is given in Table 1.

**Table 1:** Demographic information for the patients population

Subject code	Prosthetic knee	Amputation side	Age (years old)	Height (cm)	Weight (kg)	Amputation cause	K level
22_F	Genium	L	49	178	100	Trauma	4
26_F	Genium	L	29	172	65	Trauma	4
27_F	Genium	R	59	170	80	Trauma	4
28_F	Genium	R	44	192	103	Trauma	4
30_F	Genium	L	48	174	90	Trauma	4
51_F	Genium	L	69	175	76	Trauma	4
48_F	Genium	L	30	178	95	Trauma	4
16_F	C-Leg	R	56	175	72	Trauma	4
17_F	C-Leg	L	74	170	85	Trauma	4
20_F	C-Leg	R	72	163	87	Trauma	4
34_F	C-Leg	R	52	178	115	Trauma	4
35_F	C-Leg	L	53	183	67	Trauma	4
36_F	C-Leg	R	39	175	95	Trauma	4
52_F	C-Leg	L	36	170	70	Trauma	4
1_F	Polycentric mechanical	R	68	170	78	Trauma	4
2_F	Polycentric mechanical	R	62	186	91	Trauma	4
5_F	Polycentric mechanical	L	57	171	82	Trauma	4
6_F	Polycentric mechanical	L	52	178	115	Trauma	4
7_F	Polycentric mechanical	L	63	165	78	Trauma	4

For the analysis, the right side of the control subjects was compared with the sound side of the patients, while the left side was compared with the prosthetic one. This choice has been made in order not to have an effect of the dominance, since amputation side has no relationship with the dominant side in patients, an arbitrary side has been chosen for the comparison with healthy people. Participants gave written informed consent to procedures approved by the local Ethics Committee (Rome branch of the INAIL Prosthesis Center, at the CTO “A. Alesini” in Rome), in conformity with the Declaration of Helsinki regarding the use of human participants in research.

### Experimental protocol

The experiments were performed in the motion analysis lab of the Prostheses Centre of the Italian Workers Compensation Authority (INAIL) at the CTO Andrea Alesini Hospital of Rome, equipped with 6 cameras (BTS SMART DX 6000). All the reflective markers were placed according to the Davis protocol [22]. In order to calculate thigh, shank and foot elevation angles, only the markers attached to the skin overlying the Great Trochanter, lateral Femoral Condyle, Fibula Head, Lateral Malleolus, Heel and fifth Metatarsal Head were used. Due to the absence of clear anatomical landmarks, amputated limb markers were placed over specific points that were symmetrical with respect to the homologous marker position on the non-amputated side [2, 19], targeting the fixed parts of the prosthetic device, to easily identify the corresponding homologous segments on the amputated side without affecting the calculation of the kinematics. The 3-D coordinates of the aforementioned markers have been projected onto the sagittal plane of the subject, identified by the vertical direction and the direction of the speed of the Center of Mass, calculated as the triangle formed by bilateral Iliac Spinae and the sacrum markers. [16]. Then, the projected coordinates were exploited to calculate the angles with the vertical direction using trigonometry rules, as detailed in the following:

1 $$\theta =\text{arctan}\left(\frac{{x}_{distal}-{x}_{proximal}}{{y}_{distal}-{y}_{proximal}}\right)$$

where x and y denote the antero-posterior and the vertical coordinates of the markers projected in the sagittal plane.

Each participant performed 10 walking trials along a 9 m long walkway at a self-selected comfortable speed (1.2 ± 0.1 m/s controls and 0.9 ± 0.2 m/s, 0.9 ± 0.1 m/s and 0.9 ± 0.2 m/s for FM, FE and FB groups, respectively). Control subjects performed 10 additional walking trials at a slower speed (0.9 ± 0.1 m/s) to match the typical walking speed of people with transfemoral amputation. In the following analysis, the two walking conditions of the control subjects will be used to build two different groups, CSS and CSL, indicating self-selected and slow speeds respectively, which will be compared with the three groups of patients (FM, FE, FB).

### Data analysis

**Fig. 1:**
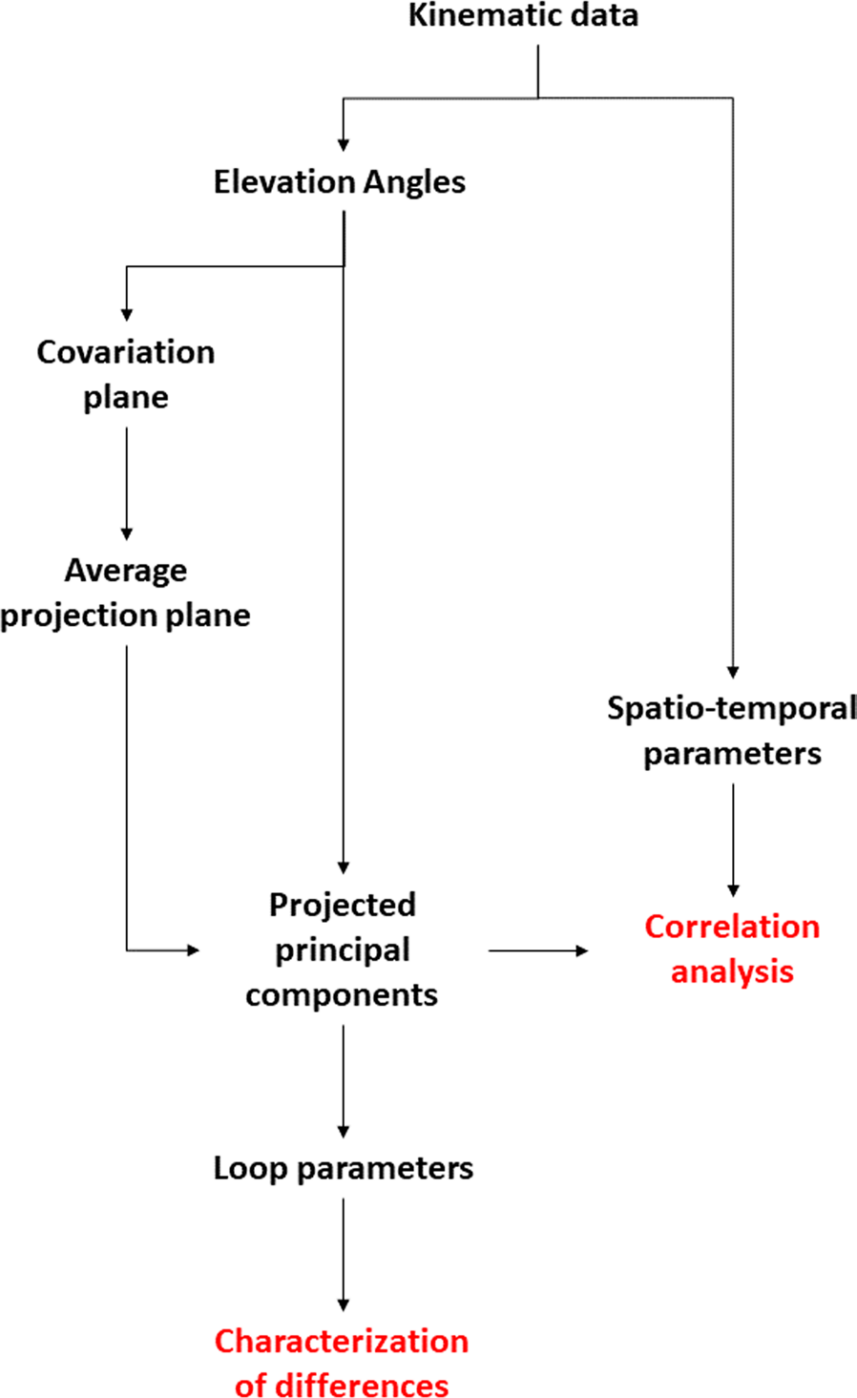
A schematic representation of the data analysis procedures involved in this work

A flow-diagram of the data analysis procedure is reported in Fig. 1. The analysis has been divided into two main parts: the first one being the analysis of the differences in the loop parameters across the different groups. The second analysis is focused on a semi-qualitative correlation analysis to test one possible biomechanical interpretation of the previous results.

All the analyses on the loop parameters have been carried out by projecting all the time courses of the elevation angles of all the subjects on a common reference plane, defined by averaging the unit vectors describing the planes of the control subjects walking at a self-selected normal speed. The projection has been realized by calculating the dot product between the vector describing the point-by-point elevation angles and the unit vectors related to the healthy subjects’ common plane.

### Validation of the common plane model

In order to evaluate the validity of the projection onto the common plane of the elevation angles of all the subjects, Variance Accounted For (VAF) values coming from this representation have been compared with the ones coming from surrogate data. VAF values have been calculated using the formula:

2 $$VAF=1- \frac{\sum {\left(M-{M}_{rec}\right)}^{2}}{\sum {\left(M\right)}^{2}}$$

where M and M_rec_ are the original and reconstructed (according to the low-dimensional model) matrices of the elevation angles.

In detail, surrogate data have been generated by transforming the original data in the Fourier domain, shuffling the phase information of the transformed data and using an inverse Fourier transform to get the surrogate data time series. The 95-percentile of the VAF values resulting from 1000 replicates of the surrogate generation has been used to define a subject-specific VAF threshold; if the VAF value coming from the projection onto the common plane is higher than this threshold, the model is supposed to be valid for the particular subject.

An additional procedure for surrogate data generation has been carried out by random shuffling the elements of the matrix describing the orientation of the plane, and repeating the same comparison with the 95-percentile of the corresponding distribution.

### Loop parameters

In order to check whether a compact description of the kinematics of lower limb elevation angles is able to yield an accurate characterization of the differences among the groups under analysis, some compact parameters have been defined starting from the projection of the elevation angles on the common covariance plane defined from the control subjects walking at a normal speed.

Regardless of the VAF values and of the surrogate data test, we computed several parameters from the loop resulting from the projection onto the common plane defined as before, as in [23], namely:

- Area: defined as the area enclosed by the loop curve (sArea and pArea for the sound and prosthetic side)
- Loop distances: defined as the linear distance between relevant gait events (heel strikes and toe offs of both legs) on the loop (sdTot__{1,2,3,4}_ and pdTot__{1,2,3,4}_ for the sound and prosthetic side and for the four gait sub-phases, respectively).
- Lengths: defined as the ratio between the length of the curve between two events and the corresponding loop distance. Values of 1 for this parameter mean that the time course of the two principal components between two events follow a straight line, minimizing the path (sLengths__{1,2,3,4}_ and pLengths__{1,2,3,4}_ for the sound and prosthetic side and for the four gait sub-phases, respectively)

### Biomechanical meaning

In order to test the biomechanical and clinical significance of the elevation angles parameters, the loop parameters have been used as input to a linear regressor that has been used to estimate all the spatio-temporal parameters and the margin of stability (MoS), defined as in [6], of both legs. In particular, the output parameters were defined as follows:

- SL: step length
- SW: step width
- StrL: stride length
- Stance, DS1, SS, DS2: relative duration of the whole stance, first and second double support and single stance phases
- Cadence: number of steps per minute
- Speed: average walking speed
- APMos__TO_ and MLMos__TO_: antero-posterior and medio-lateral MoS calculated at the contralateral toe-off.
- APMos__HS_ and MLMos__HS_: antero-posterior and medio-lateral MoS calculated at the contralateral heel strike.

For each output parameter, a different linear regressor has been implemented. In order to select the most meaningful parameters, the F values of the correlation between each input parameter and each output have been calculated using the f_regression function in the Python package sklearn. The 95-percentile of the distribution of these values has been then selected as a threshold for selecting the features; the selected subgroup of features has been defined by keeping all the features that presented at least one F value greater than the threshold, across all the outcomes.

## Results

**Fig. 2:**
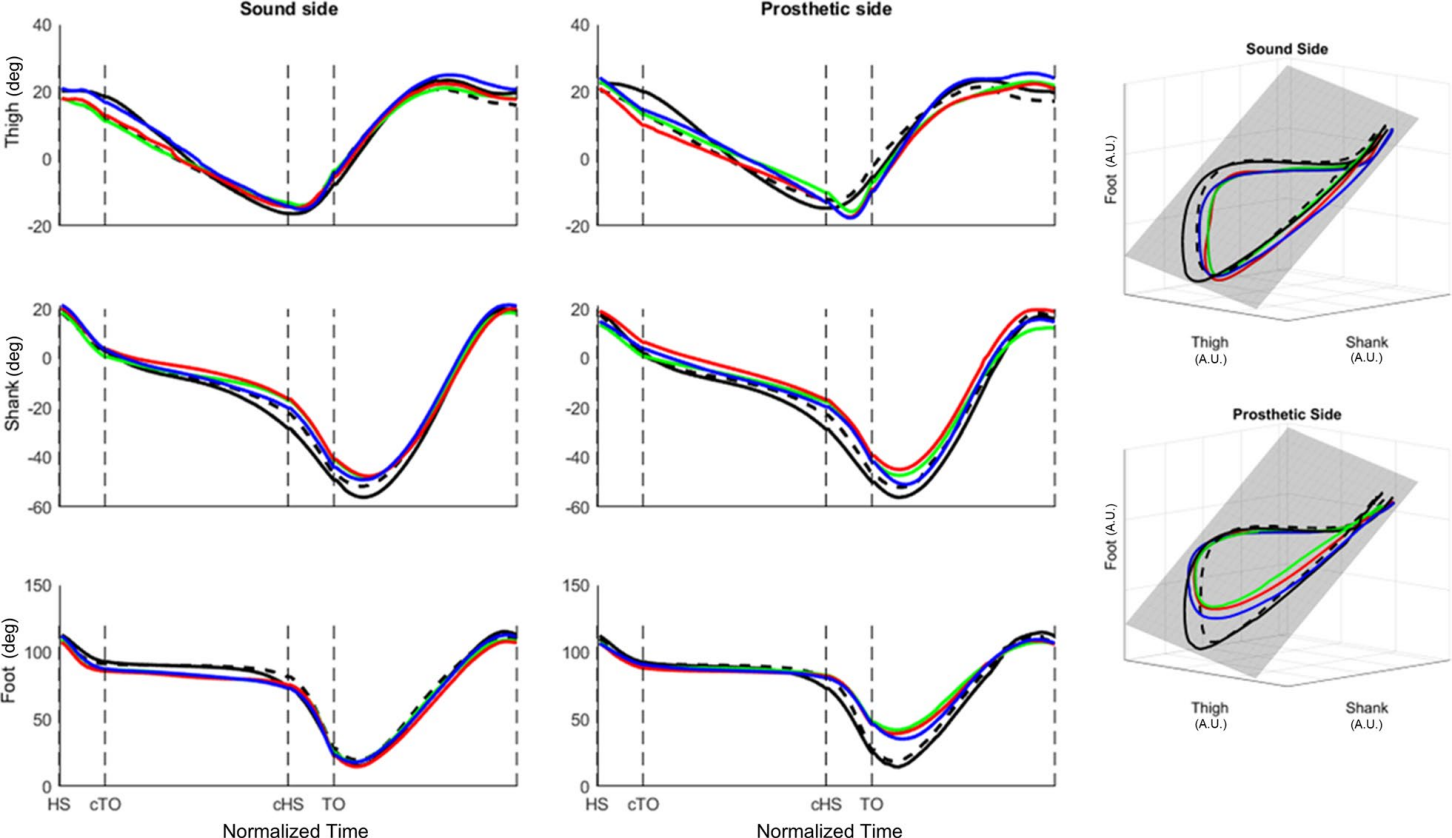
Average elevation angles time course, with the corresponding 3-D representation (solid black: control, self-selected; dashed black: control, slow; blue: FB; red: FE; green: FM; shaded grey: CSS plane of covariance). Gait cycle has been normalized to 200 samples, with a fixed 80/20 ratio between double support phases and the interposed stance and swing. Elevation angles (left) are expressed in degrees and loop projections (right) are expressed in normalized arbitrary units

The average elevation angles curves for both sides are shown in Fig. 2. In the right-hand side of the figure, the same curves are presented in the 3D space and compared with the covariance plane relative to the control subjects.

**Fig. 3:**
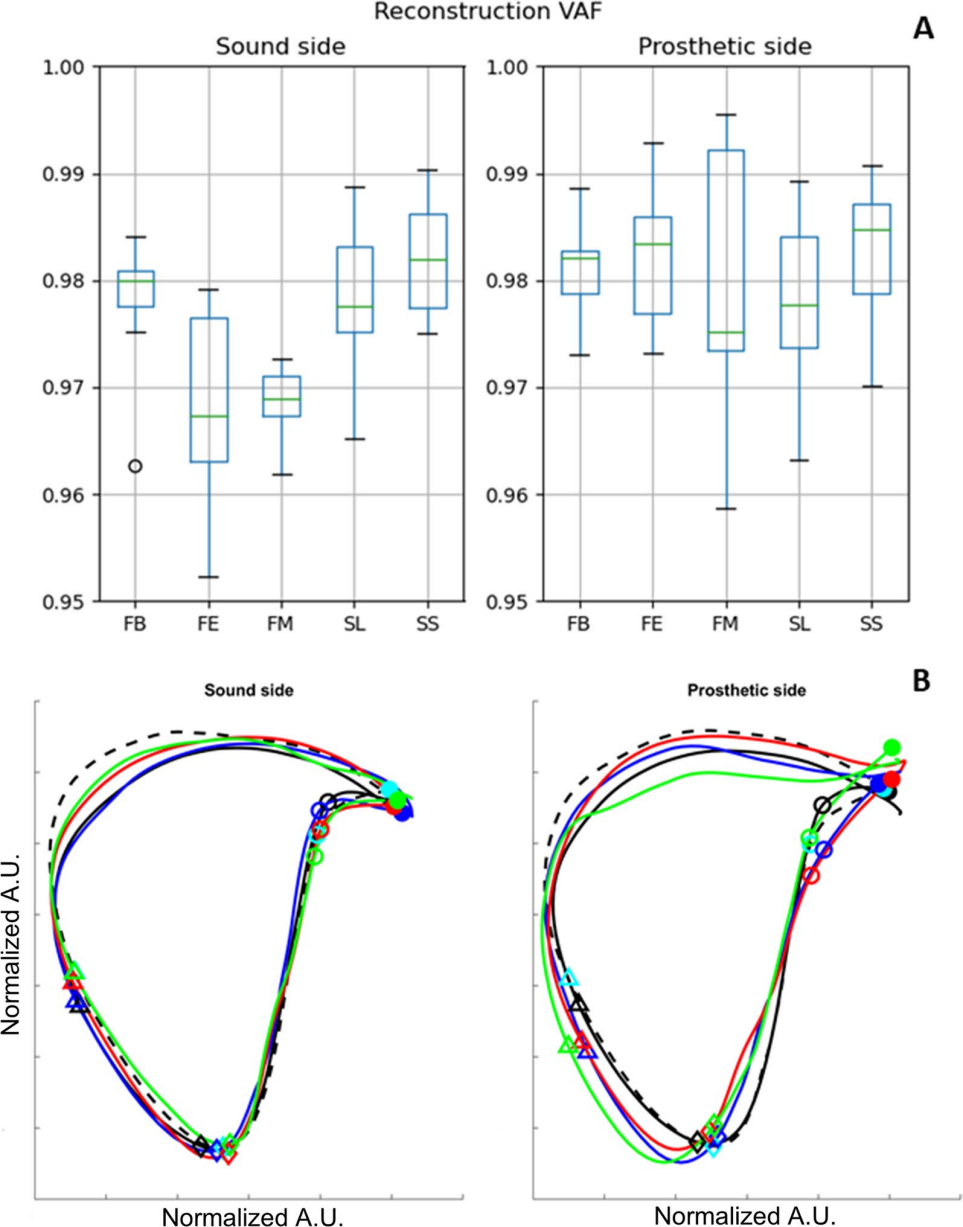
**A** VAF values for the projection onto the common plane. **B** Loops on the common plane (solid black: control, self-selected; dashed black: control, slow; blue: FB; red: FE; green: FM). Heel strike, contralateral toe-off, contralateral heel strike and toe-off have been marked with circles, diamonds, triangles, and dots respectively. Slow controls events have been marked in cyan for readability purposes. Loop projections are expressed in arbitrary units. Loop parameters are expressed in normalized arbitrary units

VAF values coming from the projection on the common plane are reported in Fig. 3A. While for the prosthetic side the results are essentially equivalent across all the groups, both FM and FE show a significant drop of VAF with respect to controls and FB on the sound side. To test equivalence of the plane, a Wilcoxon test has been carried out on the U3 component of the rotation matrix representing plane orientation [13]; no statistical differences have been found between all the pairs of groups and side, except for the prosthetic side comparison between SL subject and all the patients, considered as a whole group.

**Fig. 4:**
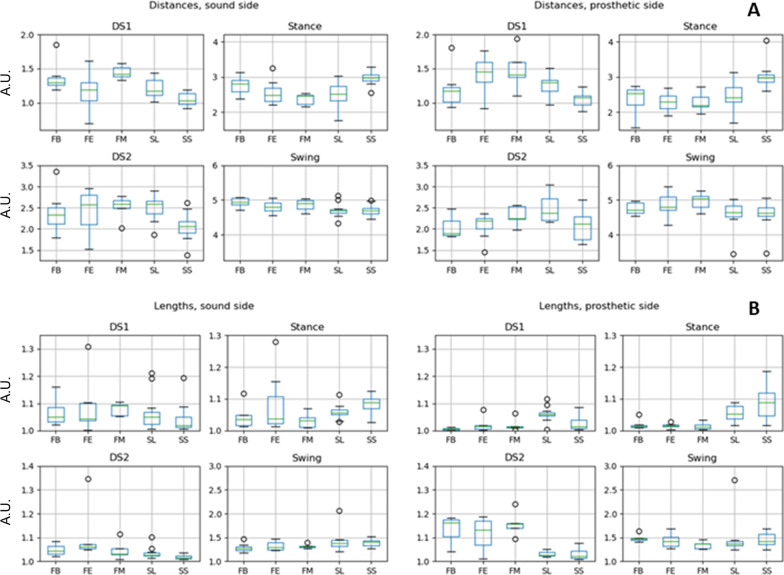
Box and whiskers plot of **A** distance parameters. **B** Length parameters

In Fig. 4A the comparison on the loop distances is given. From a general point of view, patients show differences in all the gait phases, with respect to both control groups. While these differences spread across all three groups of amputees, FB patients often show values of distances closer to the controls as compared with FE and FM.

The surrogate data coming from the random shuffling of the plane orientation suggest that the common plane model is valid for all subjects. However, the Fourier-based surrogate data show that this description is valid for the prosthetic side for all tested subjects, while on the sound one it fails to describe the coordination of 2 out of 7 FE and 1 out of 5 FM subjects.

The loops in the projection plane are presented in Fig. 3B. From the Figure, most of the qualitative differences can be identified on the prosthetic side, particularly in the configuration at the relevant gait events and on the path during the swing phase. Moreover, the most relevant deviations from the healthy patterns can be recognized in the FM data.

The results for the loop lengths are presented in Fig. 4B. Based on the loop distances, patients show significantly different parameters across the whole gait cycle. Moreover, during DS1 and Stance of the prosthetic side, amputees show length values close to 1, indicating a strict linear relationship between the two principal components of the elevation angles. Regardless of all these differences in loop distances and lengths, no difference has been found on the loop areas.

**Fig. 5:**
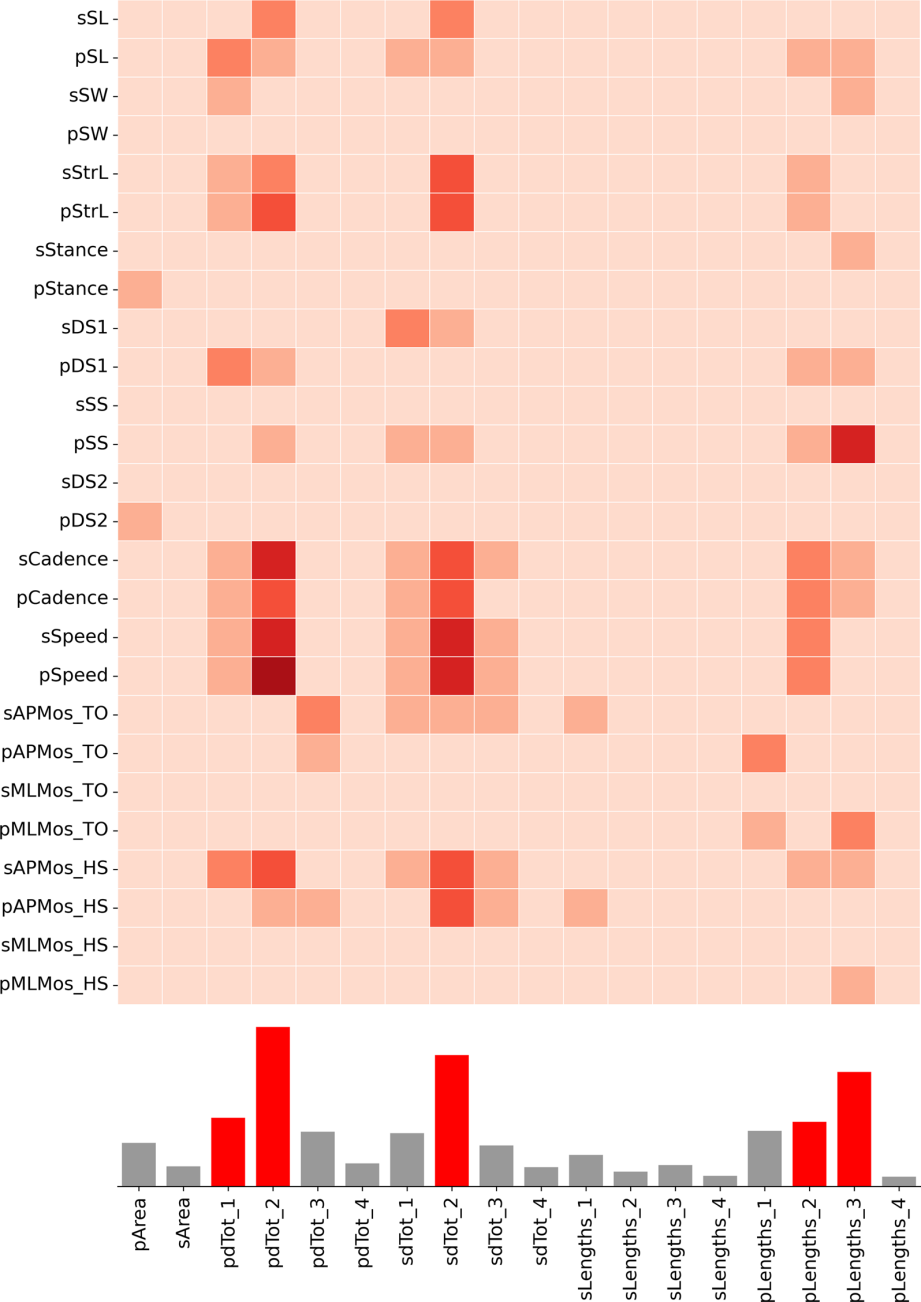
F values for the correlation between each pair of loop feature and gait parameters. Darker colours mean higher F value. Bottom row, average F value for each loop parameter; the values higher than the 95-percentile of the distribution have been marked in red

The correlation analysis (Fig. 5) show that most of the predictive information contained in the loop parameters refer to the stance phase. Specifically, the predictive parameters (i.e. the parameters with an F score higher than the 95-percentile) have been found to correspond to the linear distances between events of the stance phase on both sides, the lengths of two of the three stance sub-phases on the prosthetic side and the loop area on the same side.

The values of f-score for the correlation across all the parameters are shown in the heatmap of Fig. 5. From these data, it can be inferred that the stronger relationship between the loop parameters and the spatiotemporal characteristics of gait are related to speed and cadence, with slightly weaker correlations with the step and stride lengths. Regarding the margin of stability, only the antero-posterior MoS at the contralateral heel strike is slightly correlated with the loop parameters.

Ranking the predictive parameters by their regression F score, the area of the prosthetic loop is the least significant, with average F score values closer to the 95-percentile threshold, while the most informative parameter is the loop distance related to the single stance sub-phase of gait, on both sides.

## Discussion

This study highlights some crucial features of the modular motor control of gait kinematics in people with transfemoral amputation. Firstly, one main result is that the validity of the common plane description is different between the sound and prosthetic side, and that the differences as a function of the kind of prosthetic knee are visible on the sound side. While these latter results might seem counterintuitive, the main hypothesis at the core of this work is that most of the adaptation and compensation mechanisms are realized by the unaffected limb, and these differences in projected VAF values on the sound side support this kind of interpretation. Moreover, the control mechanisms that are implemented in the prosthetic knees included in this study, regardless of the technology, adapt their behavior mainly based on onboard inertial measurement; this technological feature leads to a better mimicking of the physiological movement on the prosthetic side, without considering the potential alterations in the contralateral leg. This might lead to a solution in which the basic control structures of the sound limb are modified, while the prosthesis is essentially replicating some features of the healthy physiological control patterns.

**Fig. 6:**
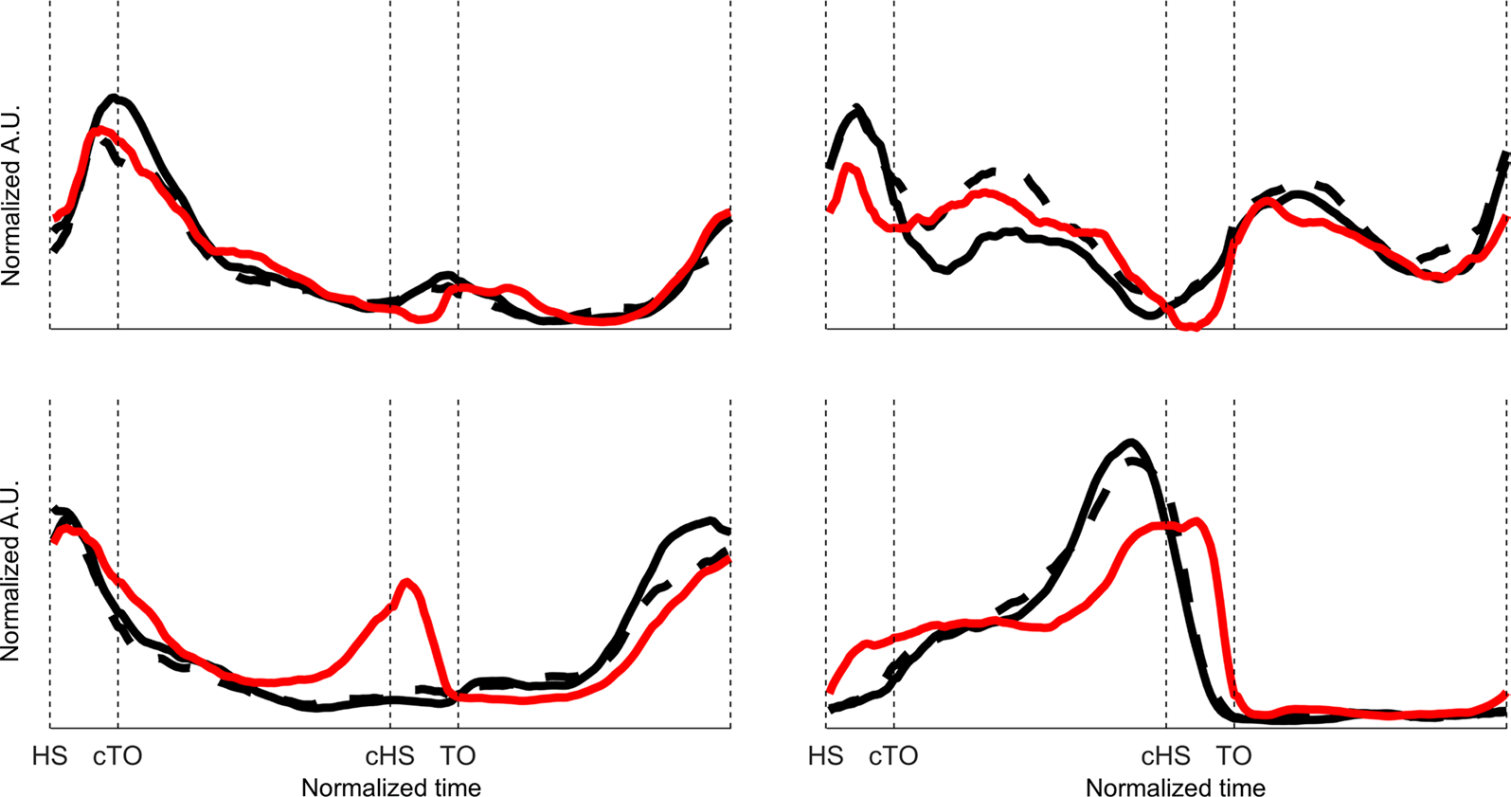
The four activation profiles for the muscle synergies characterized in [12], expressed in normalized units over an average gait cycle. Black solid: control, self-selected; black dashed: control, slow; red: patients. Activation profiles are expressed in normalized arbitrary units

The results on the loop distances, at the same time, show that while the distances between the events marking the three stance sub-phases differ among groups, the swing phase remains almost unchanged in the different groups under analysis. This indicates a different behavior of the kinematics of the leg during stance, while the leg configuration in terms of the elevation angles at its beginning and end is equivalent in amputees and control subjects. On the prosthetic side, the fact that the users of the bionic prosthesis are closer to the SS group might indicate that this kind of prosthetic knee performs better in targeting the physiological kinematic configuration at the key gait events, while the electronic and mechanical prostheses are less accurate. The same behavior cannot be identified clearly also on the sound side, particularly for the double support phases, suggesting that all the three prosthetic knees require similar compensation actions during the weight transfer phases, remarking what has been found for the muscle synergies in [12] and for general kinematic [24] and muscle co-activation [25] analyses, and confirming the general trend described in [26]. The activation profiles recorded in the aforementioned study are reported in Fig. 6 and show most of the differences in the weight transfer phases that are most relevant in the hamstrings (bottom left) and calf (bottom right) activity; this indicates that from both a kinematic and a neuromuscular point of view, modular motor control strategies are altered in prosthetic gait in a similar manner, yielding additional proof of a modular scheme also in the case of adaptation to strongly altered biomechanical demands.

The lengths of the different gait phases on the loops reflect the fact that the prosthesis behavior is strongly dependent on the hip action during stance; in DS1 and Stance the length values are close to 1, suggesting an almost linear relationship between the two principal components, up to the point in which the contralateral limb gets in contact with the ground for absorbing the body weight. These small values of the lengths in DS1 and Stance for the prosthetic limb are followed by longer paths of the two principal components in DS2; while the precise biomechanical meaning of this feature is still to be investigated, it is possible to hypothesize that this longer path with respect to the able-bodied subjects means a less smooth transition of the elevation angles during this phase. Results on the sound side, in contrast, show no relevant differences among the five groups.

The results of the correlation analysis should be considered preliminary to a deeper investigation of the biomechanical meaning of the parameters under analysis; the values for the correlations are strongly influenced by the number of subjects under analysis. Even with these limitations, some general results can be inferred. As already mentioned, swing parameters do not provide any predictive information on the spatiotemporal description of gait performance and gait stability. Moreover, most of the information is enclosed in the relative position of the events relative to the stance phase and its subdivisions; additional parameters, such as the lengths or the area, are relevant only when the prosthetic side is taken into account. This description is coherent with what has been presented with regards to the differences in the parameters themselves. The predicted parameters span spatial (step length), temporal (duration of the phases, cadence), spatiotemporal (speed) and stability parameters, suggesting that the low-dimensional description of the kinematics is able to provide information on most aspects of the gait cycle.

A limitation to this kind of analysis might be identified in the choice of using a common reference plane even when the corresponding VAF values are low; however, the presented results might play a key role in enhancing control of prosthetic devices and in this sense, having a common plane can represent a key requirement for an effective prosthesis control algorithm [27, 28]. Also by losing accuracy in the reconstruction of the original angles (i.e. projecting on a common plane), the presented results prove that it is possible to have a description that can reconstruct several important features of pathological gait.

One key result of this analysis is represented by the fact that this kind of approximated bilateral description has been able to highlight how, to enhance the prosthesis behaviour, a bigger focus must be placed on the movement of the sound limb, considering that most of the differences in terms of prosthetic device influence are to be identified in the coordination of this leg.

## Conclusions

In conclusion, even with the limitations due to smaller VAF values in the reconstruction of patients with electronic and mechanical prostheses, it is possible to use this analysis for the characterization of the burden that is required to the sound limb for generating safe and stable walking patterns; this aspect might play a key role in the definition of synthetic indicators of prosthetic gait efficiency, regardless of the accuracy in the description of the original physical signals that are used to build the model. Moreover, the parameters extracted from the kinematic patterns in a sub-dimensional space have been shown to well correlate with relevant gait biomechanical features; this latter result could be effectively exploited for controlling the behaviour of a prosthesis based on the features of two independent control signals.

## Data Availability

The datasets generated and/or analysed during the current study are not publicly available due to clinical policy but are available from the corresponding author on reasonable request.
